# Whole-genome assembly of the coral reef Pearlscale Pygmy Angelfish (*Centropyge vrolikii)*

**DOI:** 10.1038/s41598-018-19430-x

**Published:** 2018-01-24

**Authors:** Iria Fernandez-Silva, James B. Henderson, Luiz A. Rocha, W. Brian Simison

**Affiliations:** 1 Institute for Biodiversity Science and Sustainability, California Academy of Sciences, San Francisco, USA; 2 Department of Genetics, Biochemistry and Immunology, University of Vigo, Vigo, Spain; 3 Center for Comparative Genomics, California Academy of Sciences, San Francisco, USA; 4 Department of Ichthyology, California Academy of Sciences, San Francisco, USA

## Abstract

The diversity of DNA sequencing methods and algorithms for genome assemblies presents scientists with a bewildering array of choices. Here, we construct and compare eight candidate assemblies combining overlapping shotgun read data, mate-pair and Chicago libraries and four different genome assemblers to produce a high-quality draft genome of the iconic coral reef Pearlscale Pygmy Angelfish, *Centropyge vrolikii* (family Pomacanthidae). The best candidate assembly combined all four data types and had a scaffold N50 127.5 times higher than the candidate assembly obtained from shotgun data only. Our best candidate assembly had a scaffold N50 of 8.97 Mb, contig N50 of 189,827, and 97.4% complete for BUSCO v2 (Actinopterygii set) and 95.6% complete for CEGMA matches. These contiguity and accuracy scores are higher than those of any other fish assembly released to date that did not apply linkage map information, including those based on more expensive long-read sequencing data. Our analysis of how different data types improve assembly quality will help others choose the most appropriate *de novo* genome sequencing strategy based on resources and target applications. Furthermore, the draft genome of the Pearlscale Pygmy angelfish will play an important role in future studies of coral reef fish evolution, diversity and conservation.

## Introduction

Advances in genomic approaches addressing questions in ecology and evolution have led to a dramatic increase in the number of *de novo* whole genome assemblies of non-model organisms^1^. This, in turn, has led to the active development of numerous competing assembly algorithms and strategies that utilize data from the various emerging sequencing platforms^2^. *De novo* assemblies face many challenges, such as the presence of large genomic portions of repetitive content, including the repetitive structure near centromeres and telomeres, large paralogous gene families, and interspersed nuclear elements such as LINEs and SINEs, which often lead to fragmented assemblies. To overcome these challenges, various long read sequencing platforms have emerged, such as Single Molecule, Real-Time (SMRT) Sequencing (PacBio), nanopore sequencing (Oxford Nanopore and Nabsys), and various methods that take advantage of chromosomal structure and patterns (Illumina TruSeq Synthetic Long Read, BioNano Genomics, Dovetail’s Chicago method, and 10× Genomics). The challenges of *de novo* sequencing combined with the myriad of sequencing platforms and assembly methods present researchers with the difficult task of choosing the most appropriate strategy to meet their budgets and sequencing objectives.

**Figure 1:**
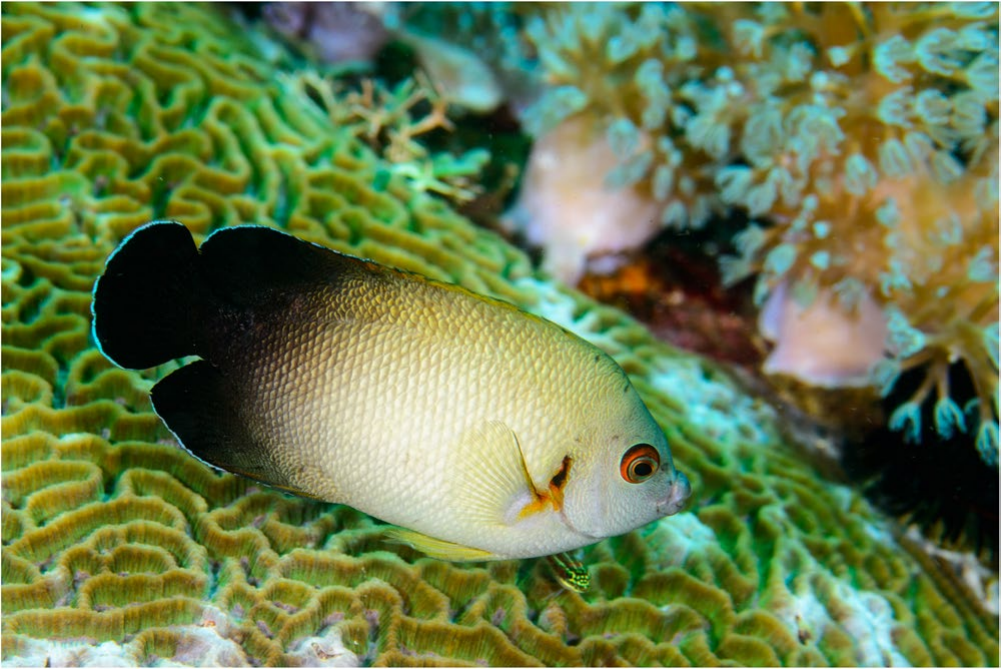
The Pearlscale Pygmy Angelfish, *Centropyge vrolikii*.

Here we present the first draft assembly of the genome of the Pearlscale Pygmy Angelfish *Centropyge vrolikii* (Bleeker, 1853) (Fig. 1). We chose a short read approach and employed a three-dataset strategy by generating overlapping deep-coverage paired-end (PE) Illumina “shotgun” read data, mate-pair libraries for mid-range scaffolding, and Chicago libraries for long-range scaffolding. We also compared various available assembly and scaffolding pipelines. A first objective was to obtain a high-quality genome assembly, then evaluate how these different methods improve assembly quality, and discuss the advantages, caveats and suitable applications of the different methods tested.

Our target species is a coral reef fish of the family Pomacanthidae (angelfishes). Although the genus *Centropyge* has a well-resolved phylogeny^3^, nominal species boundaries do not always coincide with phylogeographic breaks among genetic lineages^4^. In addition, the group of three closely related species that includes *C. vrolikii* shows extensive hybridization wherever their distributions overlap. At Christmas Island, all three species in this group (*C. vrolikii*, *C. flavissima* and *C. eibli*) hybridize^5^. Despite being indistinguishable by neutral nuclear DNA markers, these three species have strikingly different color patterns and hybrids with intermediate coloration are readily identifiable in the field. In addition, there is debate in the literature about the taxonomic status of some species in this group^6,7^. Therefore, the *C. vrolikii* genome presented here will serve as a starting point for many future studies on speciation, taxonomy, and the evolution of color and hybridization. Finally, this genome is also an important contribution to the list of marine vertebrate genomes, which currently lags far behind the list of terrestrial vertebrate genomes^1^.

## Results

### Comparison of assembly methods

A Pearlscale Pygmy Angelfish from the Philippines (CAS243847) was chosen for genome sequencing. We generated the three types of datasets outlined in Table 1: a *shotgun* dataset of overlapping Illumina 2 × 250 bp PE reads with insert size of 450 bp, two mate-pair libraries of mean insert sizes 3.4 (±0.5) kb and 5.6 (±1.7) kb and a *Chicago* library^8^.

**Table 1:** Summary of data types and Illumina read statistics used in assemblies.

Library	Read length	Illumina sequencing mode	Raw data			Filtered data		
			Read pair count	Size (bp)	Coverage*	Read pair count	Size (bp)	Coverage*
Shotgun	250 PE	1 lane HiSeq 2500 Rapid mode	166,049,777	83,024,888,500	118.61 X	124,528,871	61,983,918,428	88.55 X
Mate-pair 3.2 Kb	150 PE	1/3 lane Illumina HiSeq X SBS	136,170,869	41,123,602,438	58.75 X	91,604,453	21,700,169,234	31.00 X
Mate-pair 6.5 Kb	150 PE	1/3 lane Illumina HiSeq X SBS	135,661,062	40,969,640,724	58.53 X	33,812,494	8,032,596,441	11.48 X
Chicago	100 PE	1 lane HiSeq 2500 Rapid mode	133,206,279	26,907,668,358	38.44 X	133,206,279	26,907,668,358	38.44 X

*Genome Size = 700,000.

**Figure 2:**
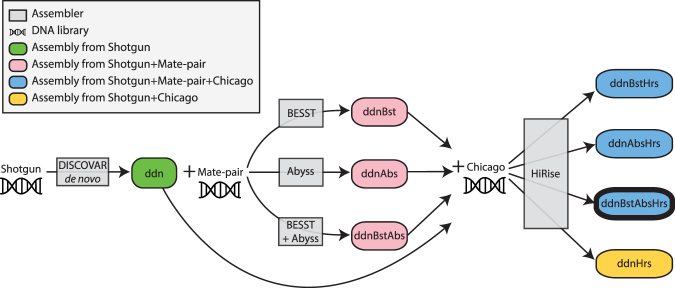
Flow chart of each of the eight candidate assemblies. Colored ovals represent each of the eight assemblies with color indicating which sources of DNA were used. The bold oval represents our highest scoring and final assembly C_vrolikii_CAS243847_v1.0.

**Figure 3:**
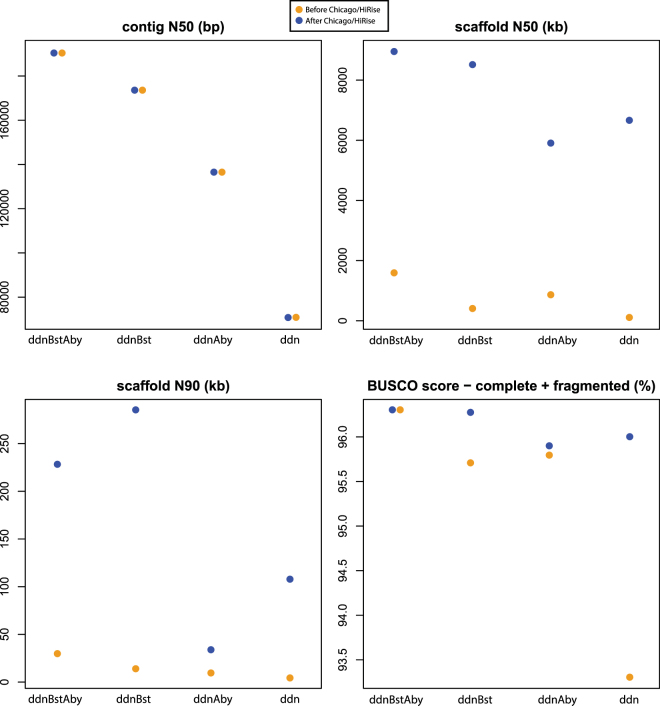
Comparison of four contiguity and accuracy statistics among the eight candidate assemblies described in Fig. 2. Contig N50, scaffold N50, scaffold N90 and the proportion of at least partial (complete and fragmented) genes present in our assembly of a set of 3,023 highly conserved single-copy orthologs (BUSCO score), considering fragments longer than 500 bp.

An aim of our work was to assess how mid- and long-range scaffolding methods improve assembly quality. An overview of the assembly pipelines and different methods applied is shown in Fig. 2. We generated and compared eight candidate assemblies (Fig. 3 and Table S1).

For the first candidate assembly, and baseline assembly for all subsequent assemblies, we used DISCOVAR de novo (ddn) (www.broadinstitute.org/software/discovar/blog) to assemble overlapping (*shotgun*) Illumina 250 bp PE reads (119× read coverage of a ~700 Mb genome) into 62,852 scaffolds, yielding an assembly length of 688.35 Mb with 0.01% gaps. Contig and scaffold N50 were 70.31 kb and 74.16 kb, respectively, and the maximum scaffold size was 940.96 kb. For the ddn assembly, an assessment of genome assembly and annotation completeness using BUSCO^9^ revealed the assembly to contain complete matches for 2,652 single-copy orthologs of a curated Vertebrata set of 3,023 (87.7%) plus partial information for 5.6% for a BUSCO score of 93.3%. In an analogous analysis of 248 core eukaryotic genes (CEGs)^10^, we found that 238 were represented (224 complete). We did not use ALLPATHS-LG to assemble the 2 × 250 bp reads because the authors of DISCOVAR de novo state “Currently the application spaces of ALLPATHS-LG and DISCOVAR are mostly complementary. Notably, ALLPATHS-LG can be used to assemble 100 base Illumina reads, and it has capabilities not yet available in DISCOVAR, including the ability to work with multiple libraries. However, DISCOVAR de novo offers the ability to create higher quality assemblies at considerably lower cost than using ALLPATHS-LG, given the appropriate data” [2 × 250 bp]. We also assembled these reads using ABYSS 2.0^11^ and SOAPdenovo 2.04^12^ each of which produced inferior contig N50 scores, 14,484 bp and 5,205 bp respectively.

Next, we used the 3.2- and 6.5-kb mate-pair libraries for scaffolding the ddn candidate assembly. We compared three different scaffolding pipelines using ABySS 1.9^13^, BESST^14,15^, and a combination of both, which generated the candidate assemblies ddnAby, ddnBst and ddnBstAby, respectively (Fig. 3). As expected, the mate-pair data improved all metrics of assembly contiguity tested (contig N50, L50, scaffold N50, L50, N90 and L90, Fig. 3 and Table S1). The assemblies generated by ABySS 1.9 produced longer scaffolds, as measured by N50, than those generated by BESST (Fig. 3 and Table S1). BESST generated more contigs greater than 1 M bp than ABySS (20 vs. 6). Scaffolding the BESST assembly with ABySS yielded the best results in terms of contiguity. A comparison of the candidate assemblies ddnBstAby and ddn, resulted in 3-, 21-, and 7-fold increases in contig N50, scaffold N50 and scaffold N90, respectively (Table S1).

However, it was the application of the Chicago method and HiRise long-range scaffolding pipeline^8^ that yielded the most dramatic increase in contiguity. For example, after the HiRise scaffolding, the scaffold N50 increased by 89.9-fold from ddn to ddnHrs and by 5.6-fold when comparing ddnBstAby to ddnBstAbyHrs. As expected from a long-range scaffolding method, the improvement on contig length (e.g. contig N50 and L50) was negligible in pre-HiRise and post-HiRise comparisons. Similarly, the CEGMA and BUSCO scores did not improve after HiRise (Fig. 3 and Table S1) likely due to the narrow margin for improvement given the high scores achieved in the pre-HiRise assemblies (95.9–96.3% BUSCO scores). To compare our assembly to previously published genomes that did not use BUSCO, we calculated both BUSCO and CEGMA scores. We used BUSCO v1 (vertebrata set) to compare previously published genomes that used the vertebrata set and BUSCO v2 with the fish specific Actinopterygii set for future comparisons (Table 2).

**Table 2:** Summary of quantitative measures for selected fish genomes. For BUSCO v1 and v2 values, the first line is the count of complete matches [count of duplicate completenes], count of fragmented, count of missing. The second line is percent of complete matches [percent duplicate completes], percent fragmented, percent missing.

Name	Scaffold N50	Contig N50	BUSCO v1 vertebrata set of 3,023	BUSCO v2 actinoptergygii set of 4,584
*Centropyge vrolikii* (ddnBstAbyHrs)	8,966,845	189,827	2,820 [219], 92, 111 93% [7.2%], 3.0%, 3.6%	4,465 [341], 40, 79 97.4% [7.4%], 0.9%, 1.7%
*Chaetodon austriacus*	170,231	38,328	2,735 [154], 117, 171 90% [5.0%], 3.8%, 5.6%	4393 [146], 90, 101 95.8% [3.2%], 2.0%, 2.2%
*Larimichthys crocea*	25,848,596	1,721,997	2,744 [118], 75, 204 90% [3.9%], 2.4%, 6.7%	4,382 [140], 52, 150 95.6% [3.1%], 1.1%, 3.3%
*Danio rerio*	53,345,113	1,263,519	2,728 [138], 99, 196 90% [4.5%], 3.2%, 6.4%	4,368 [168], 76, 140 95.3% [3.7%], 1.7%, 3.0%
*Metriaclima zebra*	3,158,421	79,912	2,790 [103], 92, 141 92% [3.4%], 3.0%, 4.6%	4,454 [95], 54, 76 97.2% [2.1%], 1.2%, 1.6%
*Oreochromis niloticus*	37,007,722	3,090,215	2,797 [102], 76, 150 92% [3.3%], 2.5%, 4.9%	4,464 [94], 42, 78 97.4% [2.1%], 0.9%, 1.7%
*Takifugu rubripes*	11,516,971	52,883	2,628 [92], 97, 298 86% [3.0%], 3.2%, 9.8%	4,419 [106], 74, 91 96.4% [2.3%], 1.6%, 2.0%

Based on highest scaffold N50/L50 scores, longest contig and scaffold lengths, and best CEGMA scores (Table S1), we chose the candidate assembly ddnBstAbyHrs for final annotation and future analyses. Although the pre-HiRise version (ddnBstAby) had a higher contig N50 than the post-HiRise version, possibly due to misassemblies corrected by the HiRise pipeline, it had a much lower scaffold N50. The quality of ddnBstAbyHrs was similar to ddnBstHrs, which had slightly better Scaffold N90/L90 values, more scaffolds longer than 1 Mbp, better BUSCO scores and a lower proportion of Ns (% of Ns). However, ddnBstAbyHrs is a good compromise between scaffold and contig N50. Henceforth, this assembly is referred to as C_vrolikii_CAS243847_v1.0, the first draft genome assembly of the Pearlscale Pygmy Angelfish.

### A high-quality draft genome of Pearlscale Pygmy Angelfish

The draft genome and annotation of *Centropyge vrolikii* (C_vrolikii_CAS243847_v1.0) was deposited in GenBank (BioProject PRJNA384789) and the Reef Genomics Database (http://reefgenomics.org). The size of the *C. vrolikii* assembly is 696.5 Mb with 1.68% gaps, distributed in 30,501 scaffolds. A list of quantitative assembly statistics is provided in Table S2. The best published estimate of genome size for the Pearlscale Pygmy Angelfish is based on cytogenetic work^16^, from which a haploid genome size of 700 Mb was calculated. We obtained a short read kmer-based genome length estimate of 606.8 Mb using preQC^17^. We also used GenomeScope^18^ to estimate genome size of 565.2 Mb and heterozygosity rate of 1.36% (Fig. S1). These numbers suggest that our assembly is complete, has no missing sequences, and that these results are an underestimate. Genome size underestimation based on kmer methods compared to assembly size is common and explained by the compression of repeats^19,20^.

The genome of the Pearlscale Pygmy Angelfish has 2n = 48 acrocentric chromosomes^21^ and is representative of the basal pattern of Percomporphaceae^22^ sensu Betancur-R *et al*.^23^. Although the length of chromosome pairs are highly variable, the average chromosome length for this genome is 29.0 Mb. The longest scaffold of C_vrolikii_CAS243847_v1.0 is 30.9 Mb, thus possibly the length of a complete chromosome. Scaffold N50 and L50 indicate that 22 of our scaffolds (scaffold L50) are longer than 8.96 Mb (scaffold N50). The contig N50 and L50 are 190 kb and 908, respectively.

### Genome validation

BUSCO identified complete information for 93.3% of the 3,023 single-copy orthologs and partial information for 3.0%, i.e. a combined total of 96.3% of the ortholog queries represented. The number of CEGs estimated using CEGMA revealed complete information for 237 of 248 CEGs and partial information for one additional CEG (95.56% complete, 95.97% at least partial).

As an additional evaluation of genome assembly quality, we assessed the mapping rate of the shotgun dataset, which was 99.4% in total, with most reads (95.6%) mapped in pairs.

### Comparison to other recently published genomes

**Figure 4:**
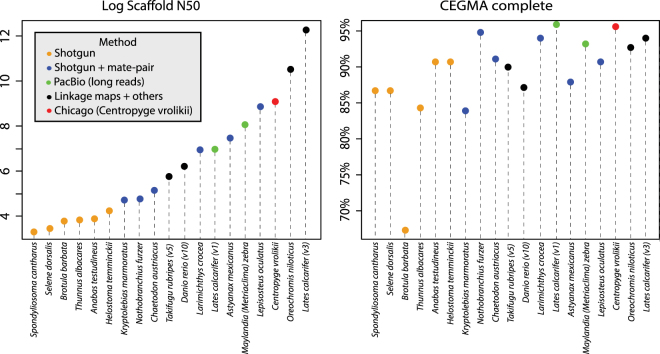
Comparison of the Pearlscale Pygmy Angelfish assembly with 18 other recently published fish assemblies. Assemblies are ranked by scaffold N50 and color coded by type of data that was utilized to generate the assembly (See also information in Table S3). Only scaffolds 1,000 bp and longer were considered for calculating scaffold N50 and CEGMA scores.

The assembly quality metrics used here are comparable to other recently published, high-quality, and scaffold-level genomes for fishes, notwithstanding that comparisons among assemblies from different species are influenced by the different genome properties and should therefore be taken with caution. Figure 4 presents a side-by-side comparison of the Pearlscale Pygmy Angelfish assembly with several other recently published fish assemblies (See also information in Table S3). We used CEGMA and BUSCO v1 (vertebrate database of 3,023 genes) for historical comparisons to published genomes and BUSCO v2 (actinopterygii database of 4,584 genes) for updated and fish specific comparisons. Our draft assembly ranks third among all representative fish assemblies compared, has better contiguity than assemblies obtained from long-reads (PacBio) and is only surpassed by two assemblies, which incorporated information from linkage maps and/or optical mapping.

Among all the fish assemblies deposited to the NCBI genome assembly database by February 24^th^ 2017, our assembly scaffold N50 ranks above all but the top seven. All assemblies with higher scaffold N50 values have applied linkage map information to their assemblies. The largest mate-pair only scaffolding assembly in the NCBI assembly database is that of the Ocean sunfish *Mola mola*, with an estimated 730 Mbp genome size, which applied five mate-pair and three PE libraries and has a scaffold N50 of ~200 kb less than the *C. vrolikii* assembly.

### Annotation

Combining homology-based and *de novo* gene prediction we identified 28,113 high confidence gene models with genes and exons representing 36.5% and 6.4% of the genome length respectively, with an average gene length of 9,049 bp (Table 3). A total of 99.2% gene models showed high confidence matches (E-value ≤ 1e-5) in the SwissProt, TrEMBL and NCBI’s non-redundant protein databases; only 236 gene models had no hits. Mean and median protein sequence length were 528 and 379 amino acids, respectively. Among the annotated genes, 97.8% were known sequences from Actinopterygii (Table S4), among which the best match hits were to sequences of *Larimichthys crocea* (49.6%), *Stegastes partitus* (15.7%), *Oreochromis niloticus* (4.5%), *Notothenia coriiceps* (3.3%) and *Dicentrarchus labrax* (2.0%), all of which belong to the same subdivision as angelfishes, Percomorphaceae (Table S4). The number of assembled scaffolds with genes was 1,202 and the number of genes per assembled scaffold ranged from 1 to 1,188, with 95% of the genes represented in 258 scaffolds (Table S5).

**Table 3:** Summary of annotation statistics for C_vrolikii_CAS243847_v1.0.

Length (bp)	Scaffold N50 (bp)	Scaffold L50	Gene Models	Gene Length (assembly %)	Average Gene Length	Repeats (assembly %)	BUSCO Complete (% of 3,023)
696,494,240	8,966,845	22	28,113	36.53%	9,049	15.95%	93.30%

### Repetitive elements and tRNA

An analysis of the repetitive element content showed that 15.94% (111 Mb) of C_vrolikii_CAS243847_v1.0 contained repeat elements (Table S6). This proportion of repeat elements is similar to what is found in other teleost genomes such as the Blacktail Butterflyfish *Chaetodon austriacus*^19^ and the Nile Tilapia *Oreochromis niloticus*^24^. We identified 1,992 tRNAs, among which 1,781 decoded the standard 20 amino acids (Table S7).

### Mitochondrion genome annotation

Two contigs of the *Centropyge vrolikii* genome (16,123 and 926 bp-long) were used to assemble an almost complete mitochondrial genome, with only 17 Ns in the D-loop control region. Since the mitochondrial contigs overlapped on both ends, assemblers for linear genomes typically leave them as separate contigs. These sequences were uploaded to the Mitos web server^25^ to validate its annotations (http://mitos.bioinf.uni-leipzig.de). The Mitos annotation confirmed 2 rRNAs, 13 coding genes and 22 tRNAs (Table S8).

### Macrosynteny with the Nile tilapia

**Figure 5:**
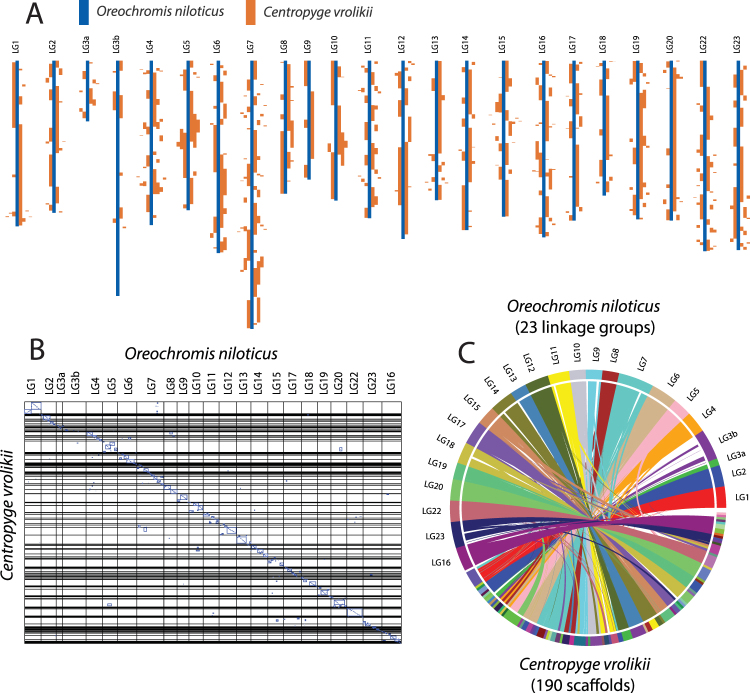
SyMAP synteny analyses between *C. vrolikii* and *O. niloticus*. (**A**) Syntenic mapping of *C. vrolikii* contigs to the *O. niloticus* chromosomes (a.k.a. linkage groups). (**B**) Whole genome dot plot. Dots represent anchors (“sequence matches”) and blue boxes indicate synteny blocks determined by the SyMap synteny-finding algorithm^27^. (**C**) Circular view of the synteny between *C. vrolikii* contigs and the *O. niloticus* chromosomes.

A synteny analysis using SyMAP vers. 4.2^26,27^ revealed 372 total shared syntenic blocks with 100 inverted, representing 92% of the *Centropyge vrolikii* and 90% of the Nile Tilapia (*Oreochromis niloticus*) contig lengths that were included in the analysis (contigs > 200,000 bp for *C. vrolikii* and all 23 linkage groups for *O. niloticus*). Of the 34,085 homologous sequences (anchors), 79% were included in syntenic blocks and more than 75% intersected annotations. Linkage groups LG3a and LG3b of tilapia are considered by Conte *et al*.^28^ to be a single chromosome (LG3) and the largest at 68.55 MB. *Centropyge* had very few syntenic matches to LG3, covered less than 15% of LG3b, and less than 21% for LG3 (LG3a and LG3b combined) (Fig. 5). All but one of the remaining tilapia linkage groups had more than 70% coverage (LG23 = 64%) with a mean coverage of 81.5%.

## Discussion

The Pearlscale Pygmy Angelfish genome assembly has one of the highest contiguity and gene completeness among fishes obtained *de novo*. Contiguity of genome assemblies not only favor completeness of assemblies, but is beneficial to analyses of structural variation and linkage. To the best of our knowledge, only chromosome-level assemblies for which linkage map information was applied, like the Nile Tilapia and the Asian Sea Bass (*Lates calcarifer*), outperform our contiguity metrics. Whereas linkage-mapping is undoubtedly a very powerful resource for assembly improvement, its application is limited to organisms that can be bred in captivity, which represent a minimal proportion of the 34,274 currently described fishes^29^. In contrast, applying the long-range scaffolding (“HiRise”) method required a moderate amount of DNA without any previous genomic information. Relative to PacBio, this approach produced a very high-quality assembly at a moderate cost. It is noteworthy that our assembly has higher metric scores than the few fish assemblies constructed thus far using PacBio long reads (Fig. 4).

The angelfish genome, like other teleosts, contains an elevated number of ohnologs (gene duplicates derived from genome duplication^30^) associated with the teleost specific whole genome duplication event (TS-WGD^31^). And like many marine organisms, the angelfish has a large effective population size resulting in a high heterozygosity rate of 1.36% (GenomeScope^18^). Both of these features present significant challenges to whole genome assemblies. The success of the Chicago/HiRise long-range scaffolding method in overcoming these challenges for the angelfish demonstrates a broad utility to the most diverse group of vertebrates on Earth.

Does long-range scaffolding preclude the need for constructing mate-pair libraries as suggested by Putnam *et al*.^8^? Our analyses suggest this is not the case. The assemblies that combined mate-pair and Chicago data (Fig. 2) were more contiguous than those that bypassed the construction of mate-pair libraries. This is reflected in a 1.3-fold increase in scaffold N50 when comparing candidate assembly ddnBstAbsHrs (the best of the assemblies that combined mate-pair mid-range scaffolding with Chicago long-range scaffolding) and the candidate assembly ddnHrs (which applied long-range scaffolding to an assembly constructed only with overlapping shotgun data; Fig. 2). However, the major improvement of contiguity is in the mid-size range (100 kb–1 Mb). The addition of HiRise assembly to the ddn assembly achieved a scaffold N90 of 107,415. The addition of mate-pair libraries to this assembly increased scaffold N90 to 227,852 for ddnBstAbsHrs and to 285,062 for ddnBstHrs.

Budgetary decisions, such as using only mate-pair or Chicago data in order to scaffold a short-read assembly, will depend on the intended applications of the assembly. Macrosynteny comparisons or any inferences about linkage benefit from assemblies producing long scaffolds^8^, ideally close to the length of the chromosomes or chromosome arms (in the 10 Mbp range for most animal genomes). However, if the assembly is to be used to identify the function of genes linked to loci of interest, such as gene expression experiments, genotype-phenotype association studies or screens of genomic divergence, having most of the genome in contigs above 25 kb is beneficial^2^. Other target applications may fall somewhere in between.

Measuring the accuracy and quality of a genome assembly is important for its use in future studies. Direct measures of accuracy require the use of independent evaluations, for example, sequencing BACs or acquiring optical mapping information. However, there are also indirect methods that can evaluate accuracy, such as estimating the proportion of curated sets of single-copy orthologs present in the assembly (BUSCO or CEGMA scores), estimating the mapping rate of DNA reads, comparing annotation metrics with other closely related genomes or analizing synteny.

Our analyses of single-copy orthologs revealed high BUSCO v1 Vertebrata dataset scores in our final assembly with 93.3% of the queries identified as complete and 96.3% at least partial; and even higher for the fish-specific BUSCO v2 Actinopterygii dataset with scores of 97.4% complete and 98.3% at least partial. These values outperform most of the genome assemblies available for fishes to date. It is noteworthy that our candidate assembly ddn, obtained with only overlapping short reads, had very high scores, with 87.7% complete and 93.6% at least partial matches of the BUSCO Vertebrata dataset queries; and 93.5% complete, 97.1% at least partial, for the BUSCO v2 Actinopterygii dataset. Our analyses suggest that the use of PE 250 bp reads in combination with the DISCOVAR de novo assembler was key in obtaining a highly accurate baseline candidate assembly for subsequent scaffolding and contributed to the overall success of producing a high-quality genome. ABySS 1.9 and BESST scaffolders were chosen based on comparisons with DISCOVAR denovo in Jackman *et al*.^32^. Our data further suggests that combining both BESST and ABySS 1.9 to our mid-range scaffolding pipeline further improved the baseline assembly. Along with effective scaffolding, the iterative approach used by BESST to incorporate the mate-pair libraries performed well in extending the length of contigs. The use of the ABySS 1.9 scaffolder resulted in large increases in the N50 through N90 metrics for both contig and scaffold results, increased the length of the longest scaffold, and improved both CEGMA and BUSCO scores. All values improved further after long-range scaffolding using the Chicago library.

A comparison of the annotation statistics obtained in closely related genomes is also an indirect measure of the assembly quality. Our assembly included 28,113 genes (36.53% of the genome length), similar to the values obtained for recently published assemblies of other percomorph fishes, 28,926 genes in the Blacktail butterflyfish *Chaetodon austriacus*^19^, 25,401 in the yellow croaker *Larimichthys crocea*^33^, 22,184 in the Asian seabass *Lates calcarifer*^34^ and 26,895 in the African cichlid *Metriaclima zebra*^24^. At least 15.94% of our assembly length has repetitive regions, a proportion that is similar to what was found in the percomorph assemblies listed above (17.86–18.60% of genome length characterized as repetitive), except for the more repeat-rich assembly of the African cichlid (28.07% in repetitive regions).

If we assume synteny among closely related genomes, investigating collinearity of genes among scaffolds of different assemblies can also provide an indication of its accuracy. Teleost genomes have been repeatedly shown to be highly syntenic^35^. A comparison of the Pearlscale Pygmy Angelfish (2n = 48) and the chromosome-level assembly of the Nile Tilapia (2n = 44) indicates elevated levels of macrosynteny, as expected. This is true for most of the Nile Tilapia genome, except for the linkage group LG3b, for which only 15% of the sequence had a syntenic match with the angelfish genome. The elevated proportion of repetitive sequences reported for the LG3b^28^ may account for this pattern, as repetitive regions are not expected to be orthologous and assembling genomic regions with high numbers of repeats is difficult. It is also noteworthy that the LG3b of the Nile tilapia genome contains a newly discovered XY sex determination region^28^. Sex is determined in the Nile Tilapia by a chromosomal system, while angelfishes are protogynous hermaphrodites for which the development of male gonads is under hormonal control influenced by social interactions.

In summary, we have shown that a combination of mid- and long-range scaffolding is a powerful strategy for obtaining a highly accurate and contiguous genome *de novo*. The angelfish genome will be a valuable tool for the study of the evolution of coral reef fishes.

## Materials and Methods

### Data acquisition

We obtained DNA sequences from a specimen of *Centropyge vrolikii* from the Philippines (CAS243847) following three different methods.

#### Shotgun

We isolated genomic DNA from a liver biopsy using Qiagen DNeasy Blood & Tissue extraction kit following the manufacturer’s protocol. We prepared shotgun libraries using Illumina’s TruSeq - PCR free library preparation kit using the ‘with-bead pond library’ construction protocol described by Fisher *et al*.^36^ modified as described in (https://software.broadinstitute.org/software/discovar/blog/?page_id=375), with a ca. 450 bp insert size. We sequenced the shotgun library in a single lane of an Illumina HiSeq 2500 Rapid mode to obtain ca. 166 million 250 bp PE reads (2 × 250). We used FastQC^37^ to check for overall quality of the data and used Trimmomatic *vers*. 35^38^ to trim adaptors (see Table S9 for adaptor sequences), crop base 249, and remove reads shorter than 100 bp. We also used Skewer *vers*. 0.1.127^39^ to remove adaptor fragments.

#### Mate-pairs

Using as starting material a mixture of muscle, liver and brain, we prepared two size selected mate-pair libraries following Illumina’s Nextera Mate Pair DNA Library Preparation protocol with 2–3 kb and 6–8 kb insert sizes. We sequenced each in 1/3 of an Illumina HiSeq X SBS lane to obtain approximately 136 million 150 bp PE reads (2 × 150 bp). We removed adaptor sequences using Trimmomatic and the TruSeq3-PE adaptor file and removed reads shorter than 40 bp. We also used Nxtrim to categorize the reads according to the orientation and adapter location and trim the circularizing adaptor^40^.

#### Chicago

We used a biopsy of brain tissue and obtained high molecular weight DNA using the protocol described in Putnam *et al*.^8^. A Chicago library^8^ was prepared by Dovetail Genomics. Approximately 500 ng of high molecular weight genomic DNA (>50 kbp mean fragment size) was reconstituted into chromatin *in vitro* and fixed with formaldehyde. Fixed chromatin was digested with DpnII, the 5′ overhangs were filled in with biotinylated nucleotides and free blunt ends were ligated. After ligation, crosslinks were reversed and the DNA purified from protein. Purified DNA was treated to remove biotin that was not internal to ligated fragments. The DNA was sheared to ~350 bp mean fragment size and sequencing libraries were generated using NEBNext Ultra enzymes and Illumina-compatible adapters. Biotin-containing fragments were then isolated using streptavidin beads before PCR enrichment of the library. The libraries were sequenced on an Illumina HiSeq 2500 to produce a total of 133 M 2 × 100 bp PE reads.

All experimental protocols with live animals were carried our in accordance with relevant guidelines and regulations and approved by the California Academy of Sciences’ Institutional Animal Care and Use Committee (CAS IACUC Approval # 2013–3).

### *De novo* assembly

#### Building contigs

Using DISCOVAR *de novo* (ddn) *vers*. 52488 (https://www.broadinstitute.org/software/discovar/blog), which incorporates its own quality filtering and error-correcting algorithms, we assembled the adapter-trimmed shotgun dataset.

#### Scaffolding

We used the mate-pair datasets to scaffold the contigs obtained by DISCOVAR *de novo*. The BESST and ABySS scaffolders were recently shown to perform well when scaffolding DISCOVAR *de novo* assemblies^11^. In order to evaluate the effect of the different scaffolding algorithms on assembly quality, we used three pipelines (Fig. 2):

1. Bias Estimating Stepwise Scaffolding Tool (BESST)^15^. Prior to scaffolding, we mapped each of the mate-pair libraries to the contig assembly using BWA^41^. The resulting mapping files (in BAM format) and the contig assembly were used as input for BESST. We set iterations and the number of witness pairs needed using the additional options -d–iter 2000000 -e 5 5.
2. ABySS-Scaffold *vers*. 1.9^13^. We provided the contig assembly and mate-pair libraries, then ran the scaffolding algorithms (steps 6–9) following Jackman *et al*.^32^. We set additionally options as j = 16 k = 200 l = 40 s = 500 S = 500–5000 N = 10 mp3k_de = –mean mp3k_n = 1 mp8k_de = –mean mp8k_n = 1.
3. BESST + ABySS. We used the mate-pair datasets to scaffold the assembly generated by BESST (method 1) using ABySS-Scaffold (steps 6–9).

#### Long-range scaffolding

We proceeded to long-range scaffolding using the software pipeline HiRise, specifically designed for the combined use of Chicago library reads (in FASTQ format), shotgun reads and a draft *de novo* assembly to assemble genomes^8^. We tested HiRise alternatively in each of the four draft assemblies described above (the contig assembly obtained by ddn and each of the three scaffold assemblies). Shotgun and Chicago library sequences were aligned to the draft input assembly using a modified SNAP read mapper (http://snap.cs.berkeley.edu). The separations of Chicago read pairs mapped within draft scaffolds were analyzed by HiRise to produce a likelihood model, and the resulting likelihood model was used to identify putative misjoins and score prospective joins. After scaffolding, shotgun sequences were used to close gaps between contigs.

### Assessment of assembly quality

To obtain genome assembly statistics, for each draft assembly we removed contigs shorter than 500 bp and used the Assemblathon 2 script^2^. Contig break was set to 25 bp. For historical reasons, we also used CEGMA v.2.5^10^ to determine the proportion of a defined core set of 248 highly conserved eukaryotic genes present in the assembly to compare our genome to previously published genome that used CEGMA. For similar reasons, we used BUSCO (*vers*. 1.1b1 modified to allow species zebrafish^9^) and its Vertebrata lineage set, which uses a set of 3,023 single-copy orthologs to assess assembly completeness. Additionally, we used BUSCO v2 and used the actinopyerygii set of 4,584 orthologs for a more fish-specific assessment. As an additional evaluation of genome assembly quality, we assessed the mapping rates of the short-insert PE library (shotgun dataset) using BWA *vers*. 0.7.5^41^ with default parameters.

### Removal of contaminants and contigs of mitochondrial origin

Based on the quality statistics we chose the assembly generated by combining ddn, BESST, ABySS and HiRise (ddnBstAbyHrs) to continue with the remainder of the analyses. We compared our assembly to GenBank’s NT database using BLASTN (with eValue and output settings -evalue 0.0001 -outfmt “10 std stitle staxid”) and removed the non-vertebrate scaffolds and those scaffolds that were of mitochondrial origin. This cleaned version without contaminants and scaffolds less than 500 bp is our final assembly.

### Comparison to other recently published genomes

We selected 18 other available fish genomes released after 2015 that are representative of current state-of-the-art assembly strategies. These were classified as (1) only shotgun data, (2) shotgun data combined with mate-pair libraries and/or BACs or Fosmids, (3) long reads (PacBio) alone or in combination with shotgun data, (4) a combination of methods and linkage mapping, (5) Chicago libraries in combination with shotgun and mate-pair data. When several representative assemblies were available for a method, we chose the ones with best contiguity metrics. We obtained values of scaffold N50 and contig N50, discarding contigs or singletons shorter than 1,000 bp. We obtained the values from the publications or estimated them ourselves using a custom modification of Assemblathon 2.0 scripts^2^.

### Annotation

We first masked our assembly using RepeatMasker^42^, which used as input the *de novo* repeat annotation created by RepeatModeler (http://www.repeatmasker.org/RepeatModeler.html) and a vertebrate repeat database (RepBase; http://www.girinst.org/repbase/). To generate protein coding gene predictions, we used MAKER2 vers 3.0^43^, which incorporates EvidenceModeler^44^ into the pipeline, supported by protein homology evidence and three *ab initio* gene prediction approaches. We set MAKER2 to annotate the 2,210 scaffolds longer than 5,000 bp. For protein homology evidence, we used an ortholog database created with Ortholog-Finder http://www.grl.shizuoka.ac.jp/~thoriike/Ortholog-Finder and the protein databases of the species described in Table S10. *Ab initio* gene prediction was accomplished by (1) training SNAP with the genes found by CEGMA; (2) using Augustus specifying species Zebrafish; (3) Self-training GeneMark with our assembly.

We ran InterProScan version 5.20–59.0^45^ to identify protein domains on the gene models generated by MAKER2. The final set of gene models was compiled from the set created by MAKER2 that had a score of AED less than 1.00 or a Pfam domain was assigned by InterProScan. We assigned names to our predicted proteins and genes using BlastP (blastp eValue < 9e-5) with the SwissProt (Sep2016) database, the Trembl (Sep2016) database, and NCBI’s non-redundant protein database (Oct2016). We annotated tRNAs using tRNAscan-SE 1.3.1^46^.

### Mitochondrial genome annotation

We compared our genome against the *Centropyge flavissima* complete mitochondrion (GenBank acc. Nr. KT275257) using blastn version 2.4.0+. Two scaffolds, one of 16123 the other of 926 bases, matched with highest scores. The fasta records were aligned to KT275257 using the Geneious version 9.1.3 aligner (http://www.geneious.com)^47^. This alignment of the three records created an almost complete mitochondrion for *C. vrolikii* with only 17 Ns in the D-loop control region. We then used mapped reads to fill in the remainder of the D-loop Control Region. To validate its annotations, the assembled mitochondrion was uploaded to the Mitos web server^25^.

### Synteny with the chromosome-level Nile tilapia genome

Using SyMAP vers. 4.2^26^ we analyzed the collinearity of gene order at the whole chromosome scale (i.e. macrosynteny as per Hane *et al*.^48^) between the Pearlscale Pygmy Angelfish C_vrolikii_CAS243847_v1.0 and the Nile Tilapia O_niloticus_UMD1^28^ assemblies. To describe the synteny analyses we adopted SyMAP terms^26,27^. “Anchor” is the homologous locations between a pair of genomes. Anchors are represented by a matrix of one genome vs. another genome (Fig. 5b). If the anchors share identical order they are referred to as “conserved” or “collinear” segments, if the anchors contain small rearrangements they are referred to as “synteny blocks”. Anchors are determined by first running RepeatMasker (www.repeatmasker.org) followed by BLAT^49^ to align sequences (for more detail see Soderlund *et al*.^26^). SyMAP is primarily designed to analyze chromosome level data. Because we are pairwise comparing 190 scaffolds rather than dozens of chromosomes, we increased the allocation of memory to 256 GB RAM by editing the ‘symap’ script by increasing the memory value in the line ‘my $maxmem = “256 g”. To reduce computation time we set a cutoff of minimum sequence length for the scaffolds to be analyzed. We determined that 96% of the Pearscale pygmy angelfish sequence was on scaffolds larger than 200,000 bp, so we set the SyMAP cutoff at this value, which resulted in 190 scaffolds to be compared. We set the length cutoff at 400,000 bp for the tilapia, which allowed inclusion of the complete length of the 23 linkage groups.

### Data and materials availability

The draft genome, C_vrolikii_CAS243847_v1.0, is available on GenBank as BioProject PRJNA384789 and BioSample accession SAMN08136993. All raw sequencing data described in this study will be available via the NCBI Sequencing Read Archive (SRA number SRP126226). The mitochondrion accession number on Genbank is MF001440.

## Electronic supplementary material

Supplementary Information 
