# Supplementary material for: Whole-genome assembly of the coral reef Pearlscale Pygmy Angelfish (Centropyge vrolikii)
Source: Sci Rep. 2018 Jan 24;8:1498. doi: 10.1038/s41598-018-19430-x (PMC5784092; doi:10.1038/s41598-018-19430-x)
Supplement: Supplementary file 1 — Supplementary Information [file 41598_2018_19430_MOESM1_ESM.pdf]

SUPPLEMENTARY FIGURE AND TABLES

Whole-genome assembly of the coral reef Pearlscale Pygmy angelfish (*Centropyge vrolikii)*

Iria Fernandez-Silva,[1,2]* James B. Henderson,[1,3] Luiz A. Rocha,[1,4] W. Brian Simison[1,3]

[1]Institute for Biodiversity Science and Sustainability, California Academy of Sciences, USA
[2]Department of Genetics, Biochemistry and Immunology, University of Vigo, Spain
[3]Center for Comparative Genomics, California Academy of Sciences, USA
[4]Department of Ichthyology, California Academy of Sciences, USA

Figure S1. GenomeScope profile obtained for the *Centropyge vrolikii* genome assembly.

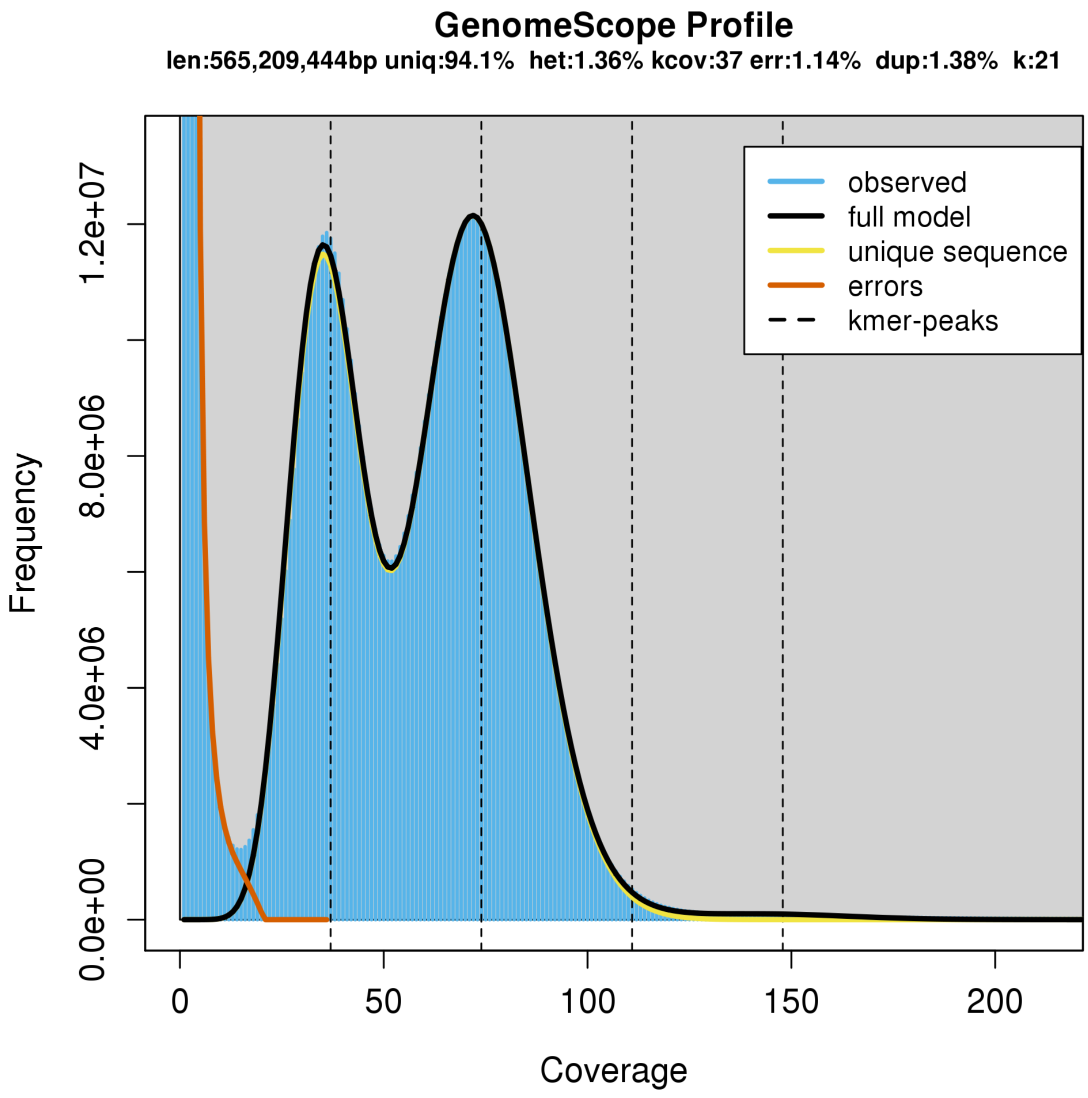

Table S1. Comparison of quality metrics among different assemblies (extended). In bold, highest scores.

Analysis	(1a) ddnBstAbyHrs	(1b) ddnBstHrs	(1c) ddnAbyHrs	(1d) ddnHrs
Number of contigs	44,768	47,968	52,665	63,739
Number of scaffolds	30,501	30,758	34,321	33,249
Contig N50 | L50	189,798 | 909*	173,258 | 987	135,936 | 1267	70,554 | 2309
Scaffold N50 | L50	**8,966,845 | 22**	8,511,577 | 24	5,895,349 | 33	6,664,922 | 28
Scaffold N90 | L90	227,852 | 185	**285,062 | 178**	34,146 | 558	107,415 | 361
Longest Contig	**1,806,211**	1,522,967	1,499,007	940,960
Longest Scaffold	**30,926,306**	30,876,611	22,727,980	27,616,649
Scaffolds > 1M	98	106	113	108
Scaffolds > 10M	20	**21**	17	16
Contigs > 100K	1728	1707	1707	1535
Contigs > 1M	25	20	6	0
% of Ns	1.68	1.03	3.07	0.44
Breaks made to assembly by HiRise	555	425	672	382
Joins made by HiRise	4993	11175	10174	23183
Gaps closed after HiRise	57	71	109	133
CEGMA (of 248) Complete | At least Partial	**237 | 238 95.56% | 95.97%**	**237 | 238 95.56% | 95.97%**	232 | 237 93.55% | 95.56%	233 | 237 93.95% | 95.56%
BUSCO v1 Vertebrata set (of 3023) Complete [Dup], Fragment, Missing	2820 [219], 92, 111 93.3% [7.2%], 3.0%, 3.7%	**2830 [182], 82, 111 93.6% [6.0%], 2.7%, 3.7%**	2794 [222], 104, 125 92.4% [7.3%], 3.4%, 4.1%	2793 [195], 109, 121 92.4% [6.5%], 3.6%, 4.0%
BUSCO v2 Actinopterygii set (of 4584) Complete [Single,Dup], Fragment, Missing	4,465 [4124,341], 40, 79 97.4% [90.0%,7.4%], 0.9%, 1.7%	4,479 [4142,337], 36, 69 97.8% [90.4%,7.4%], 0.8%, 1.4%	4,436 [4116,320], 60, 88 96.8% [89.8%,7.0%],1.3%, 1.9%	4,448 [4105,343], 50, 86 97.1% [89.6%,7.5%], 1.1%, 1.8%
	(2a) ddnBstAby	(2b) ddnBst	(2c) ddnAby	(2d) ddn
Number of contigs	44,370	47,614	52,102	63,490
Number of scaffolds	35,038	41,508	43,823	62,852
Contig N50 | L50	**191,278 | 905**	174,300 | 984	136,354 | 1264	70,311 | 2312
Scaffold N50 | L50	1,592,435 | 103	421,568 | 409	865,153 | 180	74,165 | 2168
Scaffold N90 | L90	30,433 | 1170	13,522 | 3514	9,213 | 4627	4,644 | 19170
Longest Contig	1,806,211	1,522,967	1,499,007	940,960
Longest Scaffold	10,487,947	3,708,722	7,891,921	940,960
Scaffolds > 1M	157	104	148	0
Scaffolds > 10M	3	0	0	0
Contigs > 100K	1726	1709	1708	1533
Contigs > 1M	25	20	6	0
% of Ns	1.61	0.87	2.93	0.01
CEGMA (of 248) Complete | At least Partial	236 | 238 95.16% | 95.97%	231 | 237 93.15% | 95.56%	231 | 237 93.15% | 95.56%	224 | 238 90.32% | 95.97%
BUSCO v1 Vertebrata set (of 3023) Complete [Dup], Fragment, Missing	2823 [288], 87, 113 93.4% [9.5%], 2.9%, 3.7%	2789 [336], 103, 131 92.3% [11.1%], 3.4%, 4.3%	2782 [307], 115, 126 92.0% [10.2%], 3.8%, 4.2%	2652 [324], 169, 202 87.7% [10.7%], 5.6%, 6.7%
BUSCO v2 Actinopterygii set (of 4584) Complete [Single,Dup], Fragment, Missing	4455 [4097,358], 46, 83 97.2% [89.4%,7.8%], 1.0%, 1.8%	4414 [4047,367], 73, 97 96.3% [88.3%,8.0%], 1.6%, 2.1%	4410 [4068,342], 87, 87 96.2% [88.7%, 7.5%], 1.9%, 1.9%	4287 [3935,352], 163, 134 93.5%[85.8%,7.7%], 3.6%, 2.9%

* Results before contaminants removed. Contig N50 I L50 are 189,827 I 908 for the final assembly.

Table S2. Assemblathon statistics for the best assembly (C_vrolikii_CAS243847_v1.0).

Analysis	Value
Number of scaffolds	30,501
Total size of scaffolds	696,494,240
Longest scaffold	30,926,306
Shortest scaffold	500
Number of scaffolds > 1K nt	13,097
Number of scaffolds > 10K nt	1,217
Number of scaffolds > 100K nt	232
Number of scaffolds > 1M nt	98
Number of scaffolds > 10M nt	20
Mean scaffold size	22,835
Median scaffold size	893
N50 scaffold length	8,966,845
L50 scaffold count	22
N60 scaffold length	6,231,852
L60 scaffold count	31
N70 scaffold length	3,278,851
L70 scaffold count	47
N80 scaffold length	1,575,573
L80 scaffold count	78
N90 scaffold length	229,215
L90 scaffold count	184
scaffold %A	28.63
scaffold %C	20.53
scaffold %G	20.53
scaffold %T	28.63
scaffold %N	1.68
scaffold %non-ACGTN	0
Number of scaffold non-ACGTN nt	17,924
Percentage of assembly in scaffolded contigs	0.94
Percentage of assembly in unscaffolded contigs	0.06
Average number of contigs per scaffold	1.5
Average length of break (>=25 Ns) between contigs in scaffold	818
Number of contigs	44,768
Number of contigs in scaffolds	15,398
Number of contigs not in scaffolds	29,370
Total size of contigs	684,821,215
Longest contig	1,806,211
Shortest contig	2
Number of contigs > 1K nt	27,168
Number of contigs > 10K nt	6,744
Number of contigs > 100K nt	1,728
Number of contigs > 1M nt	25
Number of contigs > 10M nt	0
Mean contig size	15,297
Median contig size	1,320
N50 contig length	189,827
L50 contig count	908
N60 contig length	135,863
L60 contig count	1,334
N70 contig length	83,048
L70 contig count	1,977
N80 contig length	35,756
L80 contig count	3,219
N90 contig length	9,185
L90 contig count	7,095
contig %A	29.12
contig %C	20.88
contig %G	20.88
contig %T	29.11
contig %N	0
contig %non-ACGTN	0
Number of contig non-ACGTN bp	17,924

Table S3. Comparison of scaffold and contig N50 for 17 recently released genomes.

Data Types	Scientific name	Common name	Family	Details	N50 scaffold length (kb)	N50 contigs length (kbs)	Genome size* (Mb)	CEGMA Complete %
1 - Only shotgun	*Spondyliosoma cantharus*	Black seabream	Sparidae		28.11	11.63	767.00	86.7
1 - Only shotgun	*Selene dorsalis*	African Moonfish	Carangidae		32.35	11.21	576.00	86.7
1 - Only shotgun	*Brotula barbata*	Bearded brotula	Ophidiidae		45.71	17.58	519.00	67.3
1 - Only shotgun	*Thunnus albacares*	Yellowfin tuna	Scombridae		46.87	16.81	836.00	84.3
1 - Only shotgun	*Anabas testudineus*	Climbing Perch	Anabantidae		50.10	18.82	576.00	90.7
1 - Only shotgun	*Helostoma temminckii*	Kissing gourami	Helostomatidae		71.66	17.06	686.00	90.7
2 - Shotgun + mid-insert size libraries	*Kryptolebias marmoratus*	Mangrove rivulus fish	Rivulidae	1 shotgun + 1 mate-pair libraries	111.54	16.10	830	83.9
2 - Shotgun + mid-insert size libraries	*Nothobranchius furzer*	African Turquoise Killifish	Nothobranchiidae	8 shotgun + 2 mate-pair libraries	119.70	8.70	1,240	94.8
2 - Shotgun + mid-insert size libraries	*Chaetodon austriacus*	Blacktail butterflyfish	Chaetodontidae	1 shotgun + 3 mate-pair libraries	170.23	21.09	712.38	91.1
	Takifugu rubripes (v5)	pufferfish	Tetraodontidae		928,938	52.88	392.37	90.32
	Danio rerio (v10)	Zebrafish	Cyprinidae		2181.22	1258.14	1,371.72	87.50
2 - Shotgun + mid-insert size libraries	*Larimichthys crocea*	Yellow croaker	Sciaenidae	1 shotgun + BAC libraries	1034.75	76.56	677.00	94.0
3 - Long reads	*Lates calcarifer (v1)*	Asian seabass	Latidae	PacBio (90x)	1066.12	1066.12	668.46	96.4
2 - Shotgun + mid-insert size libraries	*Astyanax mexicanus*	Blind cave fish	Characidae	1 shotgun +2 mate-pair libraries	1775.3	14.74	964.25	87.9
3 - Long reads	*Maylandia (Metriaclima) zebra*	African cichlid	Cichlidae	Improvement of a previous short-insert size assembly with PacBio + short reads for	3158.42	79.91	859.84	93.2

				error correction				
2 - Shotgun + mid-insert size libraries	*Lepisosteus oculatus*	Spotted gar	Lepisosteidae	1 shotgun + 2 mate-pair + 1 Fosmid	6928.1	68.30	677.00	90.7
Current Subject	*Centropyge vrolikii*	Pearlscale Angelfish	Pomacanthidae	Shotgun + 2 mate-pair + Chicago	9322.54	195.26	685.00	95.6
4 - Other	*Oreochromis niloticus*	Nile tilapia	Cichlidae	Comparison of 37 candidate assemblies Pacbio assemblies + Linkage mapping	37007.72	3090.22	1009.86	92.7
4 - Other	*Lates calcarifer*	Asian seabass	Latidae	PacBio + Optical mapping + Linkage mapping + Synteny + Transcriptome	25848.60	1722.00	586.92	94.0

Table S4. Annotation stats (extended).

**Best Matches to the 28,113 Gene Models**				
Species Matched	Species Count	Species Pct	Running Total Count	Running Total Pct
Larimichthys crocea	13932	49.56%	13932	49.56%
Stegastes partitus	4418	15.72%	18350	65.27%
Oreochromis niloticus	1264	4.50%	19614	69.77%
Notothenia coriiceps	928	3.30%	20542	73.07%
Dicentrarchus labrax	554	1.97%	21096	75.04%
Maylandia zebra	511	1.82%	21607	76.86%
Takifugu rubripes	498	1.77%	22105	78.63%
Neolamprologus brichardi	376	1.34%	22481	79.97%
Haplochromis burtoni	376	1.34%	22857	81.30%
Kryptolebias marmoratus	343	1.22%	23200	82.52%
Pundamilia nyererei	309	1.10%	23509	83.62%
Cynoglossus semilaevis	292	1.04%	23801	84.66%
Tetraodon nigroviridis	273	0.97%	24074	85.63%
Oryzias latipes	243	0.86%	24317	86.50%
Nothobranchius furzeri	241	0.86%	24558	87.35%
Austrofundulus limnaeus	237	0.84%	24795	88.20%
Fundulus heteroclitus	223	0.79%	25018	88.99%
Cyprinodon variegatus	219	0.78%	25237	89.77%
Xiphophorus maculatus	169	0.60%	25406	90.37%

Table S5. Gene count per scaffold for C_vrolikii_CAS243847_v1.0.

Scaffolds with four or more Genes					
Genes	Scaffold	Total	Total pct	Scaffold Length	Scaffold Rank
1188	Scaff_3369	1188	4.23% thru 1.	28417370	2
1029	Scaff_4262	2217	7.89% thru 2.	30926306	1
959	Scaff_244	3176	11.30% thru 3.	20474054	5
878	Scaff_8126	4054	14.42% thru 4.	19658925	7
864	Scaff_2346	4918	17.49% thru 5.	16171820	12
799	Scaff_1626	5717	20.34% thru 6.	19674103	6
789	Scaff_11368	6506	23.14% thru 7.	16883141	10
784	Scaff_6832	7290	25.93% thru 8.	22364546	3
744	Scaff_1795	8034	28.58% thru 9.	18226583	8
714	Scaff_3555	8748	31.12% thru 10.	21066610	4
691	Scaff_7489	9439	33.58% thru 11.	17471059	9
615	Scaff_7552	10054	35.76% thru 12.	16225356	11
590	Scaff_8442	10644	37.86% thru 13.	10171024	19
511	Scaff_5709	11155	39.68% thru 14.	10812371	17
505	Scaff_2923	11660	41.48% thru 15.	11043453	15
497	Scaff_5321	12157	43.24% thru 16.	10893843	16
496	Scaff_1754	12653	45.01% thru 17.	10305228	18
478	Scaff_9107	13131	46.71% thru 18.	8648348	23
435	Scaff_7831	13566	48.26% thru 19.	11531967	13
402	Scaff_10295	13968	49.69% thru 20.	9322542	21
378	Scaff_7911	14346	51.03% thru 21.	11375819	14
374	Scaff_4196	14720	52.36% thru 22.	8355712	24
346	Scaff_12645	15066	53.59% thru 23.	10093287	20
344	Scaff_396	15410	54.81% thru 24.	8216992	25
325	Scaff_2277	15735	55.97% thru 25.	6690048	29
309	Scaff_11200	16044	57.07% thru 26.	5418914	34
293	Scaff_1526	16337	58.11% thru 27.	6632909	30
289	Scaff_893	16626	59.14% thru 28.	7482531	27
273	Scaff_6820	16899	60.11% thru 29.	7194032	28
271	Scaff_7894	17170	61.07% thru 30.	5863531	32
262	Scaff_603	17432	62.01% thru 31.	8966845	22
229	Scaff_10052	17661	62.82% thru 32.	5676474	33
220	Scaff_6779	17881	63.60% thru 33.	7508011	26
218	Scaff_2680	18099	64.38% thru 34.	6231852	31
203	Scaff_7346	18302	65.10% thru 35.	3174469	48
198	Scaff_9764	18500	65.81% thru 36.	3647515	45
195	Scaff_11702	18695	66.50% thru 37.	3038085	51
186	Scaff_2160	18881	67.16% thru 38.	4997777	36
180	Scaff_4377	19061	67.80% thru 39.	4436163	38
154	Scaff_12507	19215	68.35% thru 40.	2712292	55
153	Scaff_6504	19368	68.89% thru 41.	3684984	44
145	Scaff_11774	19513	69.41% thru 42.	5364033	35
141	Scaff_12545	19654	69.91% thru 43.	3704276	43
141	Scaff_10377	19795	70.41% thru 44.	3736436	42

140	Scaff_12820	19935	70.91% thru 45.	3531232	46
139	Scaff_4286	20074	71.40% thru 46.	2022266	62
138	Scaff_10714	20212	71.90% thru 47.	4143870	39
135	Scaff_6598	20347	72.38% thru 48.	4598902	37
134	Scaff_1201	20481	72.85% thru 49.	2977680	52
123	Scaff_227	20604	73.29% thru 50.	1083656	93
122	Scaff_4649	20726	73.72% thru 51.	1600706	76
116	Scaff_2363	20842	74.14% thru 52.	2444905	58
109	Scaff_9603	20951	74.52% thru 53.	3902765	40
107	Scaff_860	21058	74.90% thru 54.	2344576	59
102	Scaff_62	21160	75.27% thru 55.	1079389	94
96	Scaff_8555	21256	75.61% thru 56.	3795186	41
95	Scaff_9046	21351	75.95% thru 57.	3278851	47
94	Scaff_11556	21445	76.28% thru 58.	2944792	53
93	Scaff_9886	21538	76.61% thru 59.	1232039	88
91	Scaff_2330	21629	76.94% thru 60.	1575573	78
89	Scaff_10670	21718	77.25% thru 61.	1226174	89
84	Scaff_11879	21802	77.55% thru 62.	2174959	61
82	Scaff_7550	21884	77.84% thru 63.	3051681	50
80	Scaff_5658	21964	78.13% thru 64.	2864775	54
80	Scaff_1000	22044	78.41% thru 65.	1956105	65
79	Scaff_6836	22123	78.69% thru 66.	1033637	97
79	Scaff_4358	22202	78.97% thru 67.	2530839	56
79	Scaff_11985	22281	79.26% thru 68.	828980	108
78	Scaff_12434	22359	79.53% thru 69.	1906456	67
78	Scaff_10568	22437	79.81% thru 70.	3160380	49
76	Scaff_5888	22513	80.08% thru 71.	2469320	57
73	Scaff_2835	22586	80.34% thru 72.	1232137	87
70	Scaff_9053	22656	80.59% thru 73.	1838603	70
70	Scaff_8168	22726	80.84% thru 74.	1872638	69
65	Scaff_999	22791	81.07% thru 75.	1508725	82
65	Scaff_2088	22856	81.30% thru 76.	1260742	86
62	Scaff_2183	22918	81.52% thru 77.	1979967	63
62	Scaff_198	22980	81.74% thru 78.	542715	129
60	Scaff_8731	23040	81.95% thru 79.	949581	105
59	Scaff_6699	23099	82.16% thru 80.	748021	116
59	Scaff_10616	23158	82.37% thru 81.	1744176	72
58	Scaff_4558	23216	82.58% thru 82.	1519816	81
58	Scaff_3107	23274	82.79% thru 83.	537027	130
57	Scaff_13071	23331	82.99% thru 84.	1597112	77
55	Scaff_7993	23386	83.19% thru 85.	1431088	84
55	Scaff_6508	23441	83.38% thru 86.	1763411	71
54	Scaff_4252	23495	83.57% thru 87.	1663882	75
54	Scaff_2380	23549	83.77% thru 88.	1539966	80
54	Scaff_10093	23603	83.96% thru 89.	1946504	66
53	Scaff_1854	23656	84.15% thru 90.	480949	134
52	Scaff_8043	23708	84.33% thru 91.	471810	136

51	Scaff_5922	23759	84.51% thru 92.	1900591	68
50	Scaff_5701	23809	84.69% thru 93.	1291407	85
47	Scaff_8445	23856	84.86% thru 94.	1483487	83
47	Scaff_6086	23903	85.02% thru 95.	771761	115
46	Scaff_2638	23949	85.19% thru 96.	689039	119
44	Scaff_2584	23993	85.34% thru 97.	1558900	79
44	Scaff_10932	24037	85.50% thru 98.	1013324	98
43	Scaff_850	24080	85.65% thru 99.	694227	118
42	Scaff_5572	24122	85.80% thru 100.	1208022	90
42	Scaff_11437	24164	85.95% thru 101.	2339612	60
41	Scaff_7241	24205	86.10% thru 102.	376434	149
40	Scaff_12934	24245	86.24% thru 103.	320422	160
38	Scaff_12402	24283	86.38% thru 104.	533226	131
37	Scaff_6788	24320	86.51% thru 105.	389769	147
36	Scaff_9050	24356	86.64% thru 106.	801361	113
36	Scaff_8307	24392	86.76% thru 107.	477057	135
36	Scaff_3237	24428	86.89% thru 108.	811996	109
35	Scaff_8367	24463	87.02% thru 109.	346979	155
35	Scaff_6870	24498	87.14% thru 110.	662612	122
34	Scaff_1262	24532	87.26% thru 111.	1733244	73
34	Scaff_11893	24566	87.38% thru 112.	1701293	74
33	Scaff_10605	24599	87.50% thru 113.	1078757	95
32	Scaff_9093	24631	87.61% thru 114.	580082	124
32	Scaff_7069	24663	87.73% thru 115.	941582	106
32	Scaff_6385	24695	87.84% thru 116.	393974	145
32	Scaff_2915	24727	87.96% thru 117.	967000	101
32	Scaff_12350	24759	88.07% thru 118.	227852	183
32	Scaff_10863	24791	88.18% thru 119.	303043	166
32	Scaff_10353	24823	88.30% thru 120.	260687	175
31	Scaff_8608	24854	88.41% thru 121.	352990	154
31	Scaff_35	24885	88.52% thru 122.	226048	184
30	Scaff_8111	24915	88.62% thru 123.	356329	152
30	Scaff_1592	24945	88.73% thru 124.	296752	167
30	Scaff_12211	24975	88.84% thru 125.	1158327	92
29	Scaff_5766	25004	88.94% thru 126.	807014	111
29	Scaff_124	25033	89.04% thru 127.	559474	127
28	Scaff_5905	25061	89.14% thru 128.	962416	103
28	Scaff_12537	25089	89.24% thru 129.	307252	165
27	Scaff_7327	25116	89.34% thru 130.	697242	117
27	Scaff_4924	25143	89.44% thru 131.	995935	99
27	Scaff_3133	25170	89.53% thru 132.	1964412	64
27	Scaff_2749	25197	89.63% thru 133.	577263	125
27	Scaff_1330	25224	89.72% thru 134.	483673	133
27	Scaff_11590	25251	89.82% thru 135.	971492	100
25	Scaff_581	25276	89.91% thru 136.	793146	114
25	Scaff_1390	25301	90.00% thru 137.	811827	110
24	Scaff_3060	25325	90.08% thru 138.	313117	163

24	Scaff_1367	25349	90.17% thru 139.	270087	172
24	Scaff_10292	25373	90.25% thru 140.	457910	138
23	Scaff_6243	25396	90.34% thru 141.	414839	141
23	Scaff_3279	25419	90.42% thru 142.	259316	177
22	Scaff_8569	25441	90.50% thru 143.	153165	207
22	Scaff_2755	25463	90.57% thru 144.	356399	151
22	Scaff_1837	25485	90.65% thru 145.	174085	199
21	Scaff_9755	25506	90.73% thru 146.	181687	195
21	Scaff_9022	25527	90.80% thru 147.	680387	120
21	Scaff_8128	25548	90.88% thru 148.	670525	121
21	Scaff_556	25569	90.95% thru 149.	157719	206
21	Scaff_4289	25590	91.03% thru 150.	179933	196
21	Scaff_3551	25611	91.10% thru 151.	1069704	96
21	Scaff_12207	25632	91.17% thru 152.	965560	102
21	Scaff_12204	25653	91.25% thru 153.	803875	112
21	Scaff_11267	25674	91.32% thru 154.	463143	137
21	Scaff_10861	25695	91.40% thru 155.	171102	201
20	Scaff_8146	25715	91.47% thru 156.	240001	180
19	Scaff_7481	25734	91.54% thru 157.	354399	153
19	Scaff_3302	25753	91.61% thru 158.	272703	171
19	Scaff_3258	25772	91.67% thru 159.	333194	159
19	Scaff_11490	25791	91.74% thru 160.	210488	186
18	Scaff_9978	25809	91.80% thru 161.	173064	200
18	Scaff_3361	25827	91.87% thru 162.	364809	150
17	Scaff_3008	25844	91.93% thru 163.	520630	132
17	Scaff_2826	25861	91.99% thru 164.	170216	202
17	Scaff_2258	25878	92.05% thru 165.	1165884	91
16	Scaff_7090	25894	92.11% thru 166.	407995	143
16	Scaff_5983	25910	92.16% thru 167.	170045	203
16	Scaff_5056	25926	92.22% thru 168.	176014	197
16	Scaff_2232	25942	92.28% thru 169.	184909	194
16	Scaff_12347	25958	92.33% thru 170.	960956	104
15	Scaff_8983	25973	92.39% thru 171.	146538	209
15	Scaff_7702	25988	92.44% thru 172.	290274	169
15	Scaff_4947	26003	92.49% thru 173.	383832	148
15	Scaff_3178	26018	92.55% thru 174.	190829	192
15	Scaff_10385	26033	92.60% thru 175.	337148	158
14	Scaff_7887	26047	92.65% thru 176.	291963	168
14	Scaff_2808	26061	92.70% thru 177.	202425	188
14	Scaff_11286	26075	92.75% thru 178.	340800	156
14	Scaff_10096	26089	92.80% thru 179.	118442	217
13	Scaff_7214	26102	92.85% thru 180.	318750	161
13	Scaff_5683	26115	92.89% thru 181.	338416	157
13	Scaff_3368	26128	92.94% thru 182.	205979	187
13	Scaff_2470	26141	92.99% thru 183.	564542	126
13	Scaff_1683	26154	93.03% thru 184.	433017	139
13	Scaff_1421	26167	93.08% thru 185.	307834	164

13	Scaff_12085	26180	93.12% thru 186.	901622	107
13	Scaff_10981	26193	93.17% thru 187.	80165	233
12	Scaff_9098	26205	93.21% thru 188.	265209	174
12	Scaff_8813	26217	93.26% thru 189.	242492	178
12	Scaff_7624	26229	93.30% thru 190.	138301	213
12	Scaff_443	26241	93.34% thru 191.	636621	123
12	Scaff_1407	26253	93.38% thru 192.	195434	191
12	Scaff_1374	26265	93.43% thru 193.	314025	162
12	Scaff_11491	26277	93.47% thru 194.	104452	221
11	Scaff_8642	26288	93.51% thru 195.	151217	208
11	Scaff_6854	26299	93.55% thru 196.	259396	176
11	Scaff_13099	26310	93.59% thru 197.	265530	173
10	Scaff_9120	26320	93.62% thru 198.	558750	128
10	Scaff_7881	26330	93.66% thru 199.	107418	220
10	Scaff_5304	26340	93.69% thru 200.	241478	179
10	Scaff_2259	26350	93.73% thru 201.	142137	211
10	Scaff_205	26360	93.76% thru 202.	198738	189
10	Scaff_12967	26370	93.80% thru 203.	419947	140
10	Scaff_11954	26380	93.84% thru 204.	93275	227
9	Scaff_9005	26389	93.87% thru 205.	197028	190
9	Scaff_7096	26398	93.90% thru 206.	143482	210
8	Scaff_245	26406	93.93% thru 207.	166127	204
8	Scaff_12195	26414	93.96% thru 208.	50073	249
8	Scaff_10821	26422	93.98% thru 209.	130271	215
8	Scaff_10136	26430	94.01% thru 210.	55353	243
7	Scaff_9569	26437	94.04% thru 211.	53668	244
7	Scaff_8182	26444	94.06% thru 212.	107429	219
7	Scaff_705	26451	94.09% thru 213.	56311	242
7	Scaff_6963	26458	94.11% thru 214.	229215	182
7	Scaff_6660	26465	94.14% thru 215.	67734	237
7	Scaff_3519	26472	94.16% thru 216.	98321	225
7	Scaff_2651	26479	94.19% thru 217.	51357	248
7	Scaff_13003	26486	94.21% thru 218.	57198	241
7	Scaff_12414	26493	94.24% thru 219.	411265	142
7	Scaff_1168	26500	94.26% thru 220.	37576	263
7	Scaff_10645	26507	94.29% thru 221.	93982	226
6	Scaff_9692	26513	94.31% thru 222.	399984	144
6	Scaff_9625	26519	94.33% thru 223.	236101	181
6	Scaff_8590	26525	94.35% thru 224.	36197	267
6	Scaff_7184	26531	94.37% thru 225.	175416	198
6	Scaff_6827	26537	94.39% thru 226.	34645	273
6	Scaff_5260	26543	94.42% thru 227.	45413	257
6	Scaff_3820	26549	94.44% thru 228.	115148	218
6	Scaff_3462	26555	94.46% thru 229.	44643	258
6	Scaff_3162	26561	94.48% thru 230.	98513	224
6	Scaff_2823	26567	94.50% thru 231.	26627	279
6	Scaff_1844	26573	94.52% thru 232.	66664	238

6	Scaff_11458	26579	94.54% thru 233.	36867	264
6	Scaff_11390	26585	94.56% thru 234.	29298	277
6	Scaff_11360	26591	94.59% thru 235.	135495	214
6	Scaff_1135	26597	94.61% thru 236.	83630	229
5	Scaff_9519	26602	94.63% thru 237.	36190	268
5	Scaff_9451	26607	94.64% thru 238.	46169	255
5	Scaff_9227	26612	94.66% thru 239.	165073	205
5	Scaff_8775	26617	94.68% thru 240.	289966	170
5	Scaff_835	26622	94.70% thru 241.	36190	269
5	Scaff_7792	26627	94.71% thru 242.	38830	261
5	Scaff_7528	26632	94.73% thru 243.	70422	236
5	Scaff_7352	26637	94.75% thru 244.	30874	275
5	Scaff_7239	26642	94.77% thru 245.	46318	254
5	Scaff_7041	26647	94.79% thru 246.	40408	260
5	Scaff_6178	26652	94.80% thru 247.	29759	276
5	Scaff_4766	26657	94.82% thru 248.	34113	274
5	Scaff_4716	26662	94.84% thru 249.	128546	216
5	Scaff_4197	26667	94.86% thru 250.	91479	228
5	Scaff_329	26672	94.87% thru 251.	53525	245
5	Scaff_2298	26677	94.89% thru 252.	212485	185
5	Scaff_12928	26682	94.91% thru 253.	100960	223
5	Scaff_11949	26687	94.93% thru 254.	189910	193
5	Scaff_11269	26692	94.95% thru 255.	49673	250
5	Scaff_10771	26697	94.96% thru 256.	73340	235
5	Scaff_10340	26702	94.98% thru 257.	52389	247
5	Scaff_10284	26707	95.00% thru 258.	390032	146
4	Scaff_9528	26711	95.01% thru 259.	34888	272
4	Scaff_8939	26715	95.03% thru 260.	36093	270
4	Scaff_8629	26719	95.04% thru 261.	36661	266
4	Scaff_8221	26723	95.06% thru 262.	43015	259
4	Scaff_8062	26727	95.07% thru 263.	17080	284
4	Scaff_7408	26731	95.08% thru 264.	49239	251
4	Scaff_7351	26735	95.10% thru 265.	19405	283
4	Scaff_7162	26739	95.11% thru 266.	139241	212
4	Scaff_6672	26743	95.13% thru 267.	80513	232
4	Scaff_5893	26747	95.14% thru 268.	83286	230
4	Scaff_5854	26751	95.16% thru 269.	47607	253
4	Scaff_5519	26755	95.17% thru 270.	21616	281
4	Scaff_525	26759	95.18% thru 271.	59344	240
4	Scaff_5091	26763	95.20% thru 272.	14251	287
4	Scaff_5087	26767	95.21% thru 273.	36695	265
4	Scaff_5003	26771	95.23% thru 274.	59688	239
4	Scaff_4530	26775	95.24% thru 275.	38141	262
4	Scaff_3837	26779	95.25% thru 276.	23464	280
4	Scaff_3312	26783	95.27% thru 277.	20211	282
4	Scaff_3269	26787	95.28% thru 278.	6678	289
4	Scaff_2505	26791	95.30% thru 279.	35638	271

4	Scaff_1975	26795	95.31% thru 280.	74133	234
4	Scaff_1536	26799	95.33% thru 281.	81241	231
4	Scaff_12158	26803	95.34% thru 282.	28331	278
4	Scaff_12117	26807	95.35% thru 283.	14857	286
4	Scaff_12007	26811	95.37% thru 284.	102030	222
4	Scaff_11518	26815	95.38% thru 285.	52451	246
4	Scaff_11194	26819	95.40% thru 286.	48554	252
4	Scaff_10973	26823	95.41% thru 287.	16800	285
4	Scaff_10530	26827	95.43% thru 288.	13325	288
4	Scaff_10301	26831	95.44% thru 289.	45941	256

Table S6. Repetitive elements.

		Number of elements*	Length (bp)	Genome length (%) **
Total bases with repetitive elements			111,098,266.00	15.94
Retroelements		64,715.00	20,162,997.00	2.89
	SINEs	11,791.00	1,405,575.00	0.2
	Penelope	628.00	94,291.00	0.01
	LINEs	68,935.00	11,607,610.00	1.67
	LTR elements	51,145.00	7,149,812.00	1.03
DNA transposons		95,677.00	6,598,062.00	0.95
DNA elements		162,424.00	25,280,976.00	3.63
Unclassified		294,013.00	5,895,936.00	7.86
Total interspersed repeats			106,797,071.00	15.33
Small RNA		5,041.00	446,821.00	0.06
Satellites		9,946.00	1,056,866.00	0.16
Simple repeats		21,279.00	3,513,771.00	0.51
Low complexity		518.00	90,343.00	0.01

* Most repeats fragmented by insertions or deletions have been counted as one element
** Genome length = 700,000,000 bp

Table S7. tRNAscan results.

tRNAscan-SE v.1.3.1 scan results

Search Mode: Eukaryotic
Searching with: tRNAscan + EufindtRNA -> Cove
Covariance model: TRNA2-euk.cm
tRNAscan parameters: Strict
EufindtRNA parameters: Relaxed (Int Cutoff= -32.1)
tRNA secondary structure

First-pass (tRNAscan/EufindtRNA) Stats:
Sequences read: 30501
Seqs w/at least 1 hit: 719
Bases read: 696494240 (x2 for both strands)
Bases in tRNAs: 565901
tRNAs predicted: 6097
Av. tRNA length: 92
Cove Stats:

Candidate tRNAs read: 6097
Cove-confirmed tRNAs: 1992
Bases scanned by Cove: 663410
% seq scanned by Cove: 0.0 %
Script CPU time: 12.88 s
Cove CPU time: 801.46 s
Scan speed: 827.8 bp/sec
Summary
----
tRNAs decoding Standard 20 AA: 1781
Selenocysteine tRNAs (TCA): 4
Possible suppressor tRNAs (CTA,TTA): 1
tRNAs with undetermined/unknown isotypes: 8
Predicted pseudogenes: 198

Total tRNAs: 1992
tRNAs with introns:
| Ser-GCT: 2 | Arg-TCT: 23 | Leu-AAG: 1 | Leu-CAA: 12 | Glu-TTC: 1 | Ile-TAT: 10 | Tyr-GTA: 42 | Trp-CCA: 1 |
Isotype (total) Anticodon Counts:
Ala (155) AGC: 76 GGC: CGC: 12 TGC: 67
Gly (109) ACC: 1 GCC: 43 CCC: 10 TCC: 55
Pro (135) AGG: 57 GGG: 32 CGG: 13 TGG: 33
Thr (91) AGT: 46 GGT: 1 CGT: 16 TGT: 28
Val (119) AAC: 60 GAC: 1 CAC: 45 TAC: 13
Ser (104) AGA: 30 GGA: CGA: 18 TGA: 21 ACT: GCT: 35
Arg (127) ACG: 38 GCG: 1 CCG: 7 TCG: 16 CCT: 40 TCT: 25
Leu (114) AAG: 31 GAG: CAG: 49 TAG: 16 CAA: 12 TAA: 6
Phe (39) AAA: GAA: 39
Asn (51) ATT: 1 GTT: 50

Lys (110) CTT: 50 TTT: 60
Asp (84) ATC: GTC: 84
Glu (154) CTC: 118 TTC: 36
His (30) ATG: 1 GTG: 29
Gln (79) CTG: 62 TTG: 17
Ile (54) AAT: 44 GAT: TAT: 10
Met (120) CAT: 120
Tyr (42) ATA: GTA: 42
Supres (1) CTA: TTA: 1
Cys (38) ACA: 1 GCA: 37
Trp (26) CCA: 26
SelCys (4) TCA: 4

Table S8. Mitochondrion genome annotation.

Name	Start	Stop	Strand	Length
trnF(gaa)	1	68	+	68
rrnS	69	1014	+	946
trnV(tac)	1020	1091	+	72
rrnL	1093	2791	+	1699
trnL2(taa)	2792	2867	+	76
nad1	2877	3836	+	960
trnI(gat)	3846	3915	+	70
trnQ(ttg)	3915	3985	-	71
trnM(cat)	3985	4054	+	70
nad2	4055	5092	+	1038
trnW(tca)	5128	5200	+	73
trnA(tgc)	5201	5269	-	69
trnN(gtt)	5272	5344	-	73
trnC(gca)	5384	5451	-	68
trnY(gta)	5452	5521	-	70
cox1	5523	7064	+	1542
trnS2(tga)	7075	7145	-	71
trnD(gtc)	7150	7221	+	72
cox2	7229	7912	+	684
trnK(ttt)	7920	7993	+	74
atp8	7995	8156	+	162
atp6	8153	8833	+	681
cox3	8836	9618	+	783
trnG(tcc)	9621	9692	+	72
nad3	9693	10040	+	348
trnR(tcg)	10042	10110	+	69
nad4l	10111	10404	+	294
nad4	10401	11771	+	1371
trnH(gtg)	11782	11851	+	70
trnS1(gct)	11852	11918	+	67
trnL1(tag)	11922	11994	+	73
nad5	12013	13824	+	1812
nad6	13833	14351	-	519

trnE(ttc)	14352	14420	-	69
cob	14425	15558	+	1134
trnT(tgt)	15566	15637	+	72
trnP(tgg)	15637	15706	-	70
Control Region	15707	16966		1260

Table S9. Adapter files used as input to Trimmomatic.

Adapter	sequence
PrefixPE/1	TACACTCTTTCCCTACACGACGCTCTTCCGATCT
PrefixPE/2	GTGACTGGAGTTCAGACGTGTGCTCTTCCGATCT
PrefixTruPE1_rc/1	AGATCGGAAGAGCGTCGTGTAGGGAAAGAGTGTA
PrefixTruPE1_rc/2	AGATCGGAAGAGCGTCGTGTAGGGAAAGAGTGTA
PrefixTruPE2_rc/1	AGATCGGAAGAGCACACGTCTGAACTCCAGTCAC
PrefixTruPE2_rc/2	AGATCGGAAGAGCACACGTCTGAACTCCAGTCAC
PE1	TACACTCTTTCCCTACACGACGCTCTTCCGATCT
PE1_rc	AGATCGGAAGAGCGTCGTGTAGGGAAAGAGTGTA
PE2	GTGACTGGAGTTCAGACGTGTGCTCTTCCGATCT
PE2_rc	AGATCGGAAGAGCACACGTCTGAACTCCAGTCAC
TruSeq_Index_4	GATCGGAAGAGCACACGTCTGAACTCCAGTCACTGACCAATCTCGTATGCCGTCTTCTGCTTG
TruSeq_Index_4_rc	CAAGCAGAAGACGGCATACGAGATTGGTCAGTGACTGGAGTTCAGACGTGTGCTCTTCCGATC
TruSeq_Univeral	AATGATACGGCGACCACCGAGATCTACACTCTTTCCCTACACGACGCTCTTCCGATCT
TruSeq_Univeral_rc	AGATCGGAAGAGCGTCGTGTAGGGAAAGAGTGTAGATCTCGGTGGTCGCCGTATCATT

Table S10. Protein databases used by Ortholog-Finder in order of priority.

Taxon	Assembly
Danio rerio	Danio_rerio.GRCz10.pep
Takifugu rubripes	Takifugu_rubripes.FUGU4.pep
Oryzias latipes	Oryzias_latipes.MEDAKA1.pep
Tetraodon nigroviridis	Tetraodon_nigroviridis.TETRAODON8.pep
Xiphophorus maculatus	Xiphophorus_maculatus.Xipmac4.4.2.pep
Gasterosteus aculeatus	Gasterosteus_aculeatus.BROADS1.pep
Lepisosteus oculatus	Lepisosteus_oculatus.LepOcu1.pep.
Oreochromis niloticus	Oreochromis_niloticus.Orenil1.0.pep
Astyanax mexicanus	Astyanax_mexicanus.AstMex102.pep
Gadus morhua	Gadus_morhua.gadMor1.pep
Poecilia formosa	Poecilia_formosa.PoeFor_5.1.2.pep
